# A Case of Undiagnosed HIV Infection in a 57-Year-Old Woman with Multiple Myeloma: Consequences on Chemotherapy Efficiency and Safety

**DOI:** 10.1155/2016/8515218

**Published:** 2016-07-20

**Authors:** I. Poizot-Martin, S. Brégigeon, C. Tamalet, R. Bouabdallah, O. Zaegel-Faucher, V. Obry-Roguet, A. Ivanova, C. E. Cano, C. Solas

**Affiliations:** ^1^Aix Marseille Université, APHM Hôpital Sainte-Marguerite, Service d'Immuno-Hématologie Clinique, 270 boulevard de Sainte Marguerite, 13274 Marseille Cedex 09, France; ^2^INSERM U912 (SESSTIM), 13006 Marseille, France; ^3^Fondation Institut Hospitalo-Universitaire Méditerranée Infection, Pôle des Maladies Infectieuses et Tropicales Clinique et Biologique, Fédération de Bactériologie-Hygiène-Virologie, CHU Timone, 264 rue Saint-Pierre, 13385 Marseille Cedex 05, France; ^4^Département d'Hématologie, Institut Paoli Calmettes, 232 boulevard de Sainte Marguerite, 13273 Marseille Cedex 09, France; ^5^Aix Marseille Université, AP-HM Hôpital de la Timone, Service de Pharmacocinétique et Toxicologie, CRO2 INSERM U911, 13385 Marseille Cedex 05, France

## Abstract

*Background*. Non-AIDS-defining cancers represent a rising health issue among HIV-infected patients. Nevertheless, HIV testing is not systematic during the initial cancer staging. Here, we report a case of HIV infection diagnosed three years after chemotherapy initiation for multiple myeloma.* Results*. A 57-year-old woman diagnosed with multiple myeloma underwent a first round of chemotherapy by bortezomib/lenalidomide and then with bortezomib/liposomal-doxorubicine/dexamethasone, with partial remission, poor hematological tolerance, and multiple episodes of pneumococcal infection. Allogenic stem cell transplantation was proposed leading to HIV testing, which revealed seropositivity, with an HIV viral load of 5.5 Log_10_/mL and severe CD4 T cell depletion (24 cells/mm^3^). Chemotherapy by bendamustin was initiated. Multidisciplinary staff decided the initiation of antiretroviral therapy with tenofovir/emtricitabin/efavirenz and prophylaxis against opportunistic infections. After 34 months, patient achieved complete remission, sustained HIV suppression, and significant CD4 recovery (450 cells/mm^3^), allowing effective pneumococcal immunization without relapse.* Conclusion*. Our case illustrates the drawback that ignored HIV infection is still causing to cancer patients receiving chemotherapy and highlights the importance of early HIV testing in oncology. A multidisciplinary approach including oncologists/hematologists, virologists, and pharmacists is recommended in order to avoid drug interactions between chemotherapy and antiretroviral drugs. Moreover, prophylactic medication is recommended in these patients regardless of CD4+ cell count at the initiation of chemotherapy.

## 1. Introduction

The incidence of non-AIDS-defining cancers in HIV-infected patients appears to be higher than the general population [1]. However, HIV infection is frequently undiagnosed in cancer patients and HIV testing is not systematic. In Europe, the fraction of HIV-infected people that ignore their HIV status has been estimated to 20 to 40% [2, 3]. Plasma cell neoplasms, of which multiple myeloma (MM) is the most common, represent 20% of all lymphoid malignancies in Europe [4] and stem cell transplantation (SCT) after systemic chemotherapy is currently the standard of care for this malignancy. Although practice is progressively evolving, HIV infection has historically been an exclusion criterion for SCT, while approved chemotherapy for multiple myeloma is frequently associated with hematological toxicity. Nevertheless, recent observations indicate that well-adapted antiretroviral treatment (cART) may improve the outcome of chemotherapy and even of SCT [5, 6]. Here, we report a case of HIV infection diagnosed three years after the initiation of systemic chemotherapy for MM in a female patient, which illustrates the consequences of ignored HIV infection on patient's outcome.

## 2. Case Presentation

**Figure 1:**
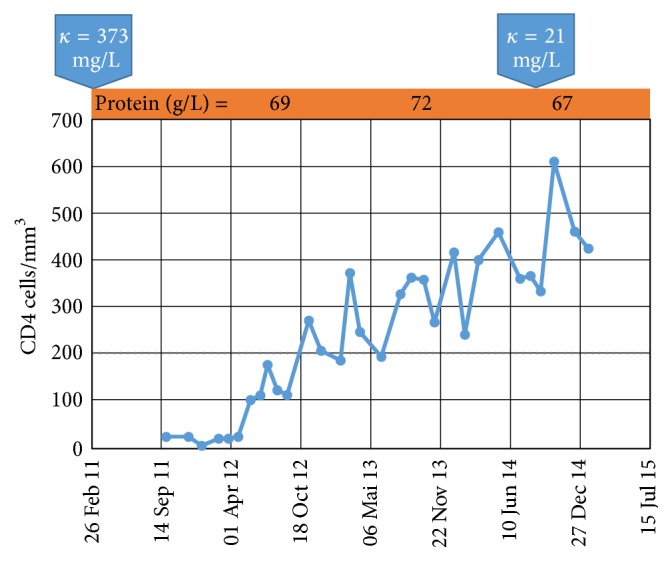
Evolution of CD4+ T cell count during and after bendamustine chemotherapy. Graph shows longitudinal evolution of CD4+ cell counts during and after bendamustine chemotherapy (from September 19, 2011, to February 29, 2012). *y*-axis = CD4 cells/mm^3^; *x*-axis = date. (Top) orange box show plasma protein quantitation (g/L).

Patient is a 57-year-old woman that presented with an inflammatory syndrome in February 2008. Clinicians diagnosed her with multiple myeloma on the basis of a positive myelography, a monoclonal IgG-*κ* gammopathy (M-protein: IgG 40 g/L; Kappa: 373 mg/L; Lambda: 195 mg/L), a *β*2-microglobulin 2 × ULN, and a diffuse infiltration of the spine detected by MRI. From March 2008 until December 2009, gammopathy was treated with bortezomib followed by lenalidomide, achieving partial remission. In June 2009, spinal MRI was normal and IgG was 30 g/L with no other abnormalities of the blood count and without renal failure. In May and December 2009, this patient received antimicrobial therapy for two episodes of pneumococcal pneumoniae. In November 2010, spinal MRI was normal. In January 2011, a combination of bortezomib, liposomal doxorubicine, and dexamethasone was initiated following worsening of gammopathy (IgG: 53 g/L), with IgA 1.74 g/L, IgM 11.03 g/L, normocytic anemia (107 g/L), platelet count 208 × 10^9^/L, creatinine 45 *μ*mol/L, normal calcemia, LDH 2 × ULN with anicteric cholestasis, *β*2-microglobuline 5 × ULN. Karyotype was normal. After 4 chemotherapy cycles with poor hematological tolerance, IgG was 25 g/L, and allogenic SCT was proposed. Of note, the patient presented with a new episode of pneumococcal pneumonia in March 2011. In August 2011, the patient underwent HIV testing before SCT; she tested positive for HIV with severe immune depression (CD4+ cell count: 24 cells/mm^3^; normal value: 600–1200) and HIV viral load 316228 copies/mL (5.5 Log_10_/mL). SCT was cancelled, and chemotherapy with bendamustin was proposed. An antiretroviral regimen combining tenofovir/emtricitabin/efavirenz and opportunistic prophylaxis (against* pneumocystis jiroveci, toxoplasma gondii,* and cytomegalovirus) was proposed by multidisciplinary staff, including a pharmacologist and virologist, to limit the risk of drug-drug interactions. After 34 months of followup, complete and sustained remission of gammopathy (IgG: 15 g/L; Kappa: 21 mg/L; Lambda: 21 mg/L; ratio 1.22) was achieved, associated with sustained HIV suppression and significant immune restoration (CD4+ T cell count: 450/mm^3^; 15%; Figure 1), which allowed for effective pneumococcal immunization without relapse.

## 3. Discussion

Here, we present a case of ignored HIV infection that severely affected the management and outcome of MM. The most likely HIV transmission route for this patient was a blood transfusion that occurred in 1978, which is 30 years prior to the diagnosis of MM and 33 years before diagnosis of HIV. Although monoclonal gammopathies of undetermined significance (MGUS) are frequent in HIV-infected patients [7, 8], our patient presented with plasma M-protein >30 g/L at diagnosis, which classifies for MM according to the International Myeloma Working Group [9]. Nevertheless, a long-term followup of MGUS patients at the Mayo Clinic revealed that around 16% of MGUS cases evolved towards a MM, with a median time of progression of 10.6 years [10]. Thus, we cannot exclude that our patient might have developed an HIV-related MGUS prior to MM.

Several studies in industrialized countries have shown that HIV-infected patients have 2 to 5 times increased risk of developing multiple myeloma compared to the general population [11–17]. Moreover, male HIV-infected patients die three times more frequently of MM compared to uninfected age-matched individuals [18]. Recently, a case-control study including 10 HIV-infected and 28 uninfected patients treated for MM showed that HIV-infected patients receiving highly active antiretroviral therapy (HAART) had longer overall and disease-free survival than uninfected controls [19]. Hence, the awareness of HIV infection and antiretroviral treatment have a proven benefit for MM patients in terms of survival.

Because invasive pneumococcal disease is a frequent complication of HIV infection, the recurrent episodes observed in this patient should have alerted physicians to the need for HIV testing. Unfortunately, missed opportunities for HIV testing are not rare [20], stressing the need to improve HIV-related recognition in all healthcare facilities and this is particularly relevant for oncologists. Indeed, systematic HIV-testing at the initial diagnosis of cancer would allow for cautious initiation of treatment with an immunosuppressive regimen for a potentially immune-compromised patient, as in this case study. Strikingly, more than 30 years after the beginning of the AIDS epidemic, patients with AIDS-defining cancers are not always tested for HIV [21]. Because antiretroviral treatment seems to have a positive impact on survival, HIV-infected patients who also have cancer should keep or initiate a cART while undergoing treatment for malignant disease [6]. However, many chemotherapeutic and antiretroviral drugs are metabolized through the cytochrome P450 (CYP) enzyme system of the liver, increasing the chemotherapy-associated toxicity or decreasing the treatment efficacy. Hence, a multidisciplinary approach to HIV-cancer care that includes physicians, hematologist/oncologists, virologists, and pharmacists is recommended to prevent the risk of drug-drug interactions and optimize clinical management [22]. In our case, the association of the antiretroviral ritonavir, a CYP450-1A2 inducer, with bendamustin, a substrate/inhibitor of CYP450-1A2, could potentially reduce the efficacy of chemotherapy. Therefore, a ritonavir-boosted protease inhibitor-based regimen was contraindicated. Interactions with other medications that are used to treat or to prevent opportunistic infections or chemotherapy side effects should also be evaluated. Indeed, prophylactic medications are recommended in these patients regardless of their CD4+ cell count at the start of chemotherapy.

Currently, scarce data are available on the prevalence of undiagnosed HIV infection among patients who present with non-AIDS-defining cancer [23]. Nevertheless, our case highlights the importance of HIV testing during the initial cancer staging. A multidisciplinary approach to HIV-cancer care that includes physicians, hematologist/oncologists, virologists, and pharmacists is particularly relevant considering the high risk of drug-drug interactions between systemic chemotherapy and antiretroviral drugs. Furthermore, prophylactic medications are recommended in these patients regardless of their CD4+ cell count at the start of chemotherapy.

## Abbreviations

SCT: Stem cell transplantation
AIDS: Acquired immune deficiency
cART: Combined antiretroviral therapy.
